# Modification of 4-(4-chlorothiophen-2-yl)thiazol-2-amine derivatives for the treatment of analgesia and inflammation: synthesis and *in vitro*, *in vivo*, and *in silico* studies 

**DOI:** 10.3389/fphar.2024.1366695

**Published:** 2024-02-29

**Authors:** Mater H. Mahnashi, Umer Rashid, Hassan Hussain Almasoudi, Mohammed H. Nahari, Imran Ahmad, Abdulkarim S. Binshaya, Osama Abdulaziz, Meshari A. Alsuwat, Muhammad Saeed Jan, Abdul Sadiq

**Affiliations:** ^1^ Department of Pharmaceutical Chemistry, Pharmacy School, Najran University, Najran, Saudi Arabia; ^2^ Department of Chemistry, COMSATS University Islamabad, Abbottabad, Pakistan; ^3^ Department of Clinical Laboratory Sciences, College of Applied Medical Sciences, Najran University, Najran, Saudi Arabia; ^4^ Faculty of Pharmacy, Bahauddin Zakaria University, Multan, Pakistan; ^5^ Department of Medical Laboratory Sciences, College of Applied medical sciences, Prince Sattam bin Abdulaziz University, Alkharj, Saudi Arabia; ^6^ Clinical Laboratory Sciences Department, College of Applied Medical Sciences, Taif University, Al-Taif, Saudi Arabia; ^7^ Department of Pharmacy, Bacha Khan University, Charsadda, Pakistan; ^8^ Department of Pharmacy, Faculty of Biological Sciences, University of Malakand, Chakdara, Pakistan

**Keywords:** analgesic, inflammation, thiazole, cyclooxygenase, LOX, *in vivo* mechanism

## Abstract

Inflammation is a protective response to a variety of infectious agents. To develop a new anti-inflammatory drug, we explored a pharmacologically important thiazole scaffold in this study. In a multi-step synthetic approach, we synthesized seven new thiazole derivatives (**5a**–**5g**). Initially, we examined the *in vitro* anti-inflammatory potentials of our compounds using COX-1, COX-2, and 5-LOX enzyme assays. After *in vitro* confirmation, the potential compounds were subjected to *in vivo* analgesic and anti-inflammatory studies. The hot plate method was used for analgesia, and carrageenan-induced inflammation was also assayed. Overall, all our compounds proved to be potent inhibitors of COX-2 compared to celecoxib (IC_50_ 0.05 μM), exhibiting IC_50_ values in the range of 0.76–9.01 μM .Compounds **5b**, **5d**, and **5e** were dominant and selective COX-2 inhibitors with the lowest IC_50_ values and selectivity index (SI) values of 42, 112, and 124, respectively. Similarly, in the COX-1 assay, our compounds were relatively less potent but still encouraging. Standard aspirin exhibited an IC_50_ value of 15.32 μM. In the 5-LOX results, once again, compounds **5d** and **5e** were dominant with IC_50_ values of 23.08 and 38.46 μM, respectively. Standard zileuton exhibited an IC_50_ value of 11.00 μM. Based on the COX/LOX and SI potencies, the compounds **5d** and **5e** were subjected to *in vivo* analgesic and anti-inflammatory studies. Compounds **5d** and **5e** at concentrations of 5, 10, and 20 mg/kg body weight were significant in animal models. Furthermore, we explored the potential role of compounds **5d** and **5e** in various phlogistic agents. Similarly, both compounds **5d** and **5e** were also significantly potent in the anti-nociceptive assay. The molecular docking interactions of these two compounds with the target proteins of COX and LOX further strengthened their potential for use in COX/LOX pathway inhibitions.

## Introduction

Inflammation is a physiological response that occurs naturally in response to a variety of infectious agents, trauma, autoimmune diseases, injury, tissue ischemia, and an imbalance. It is a process that is required for the body to heal from infection and damage. Inflammation causes biochemical changes that result in swelling, redness, pain, itching, and an increase in body temperature. However, if inflammation is not appropriately treated, leukocytes, lymphocytes, or damaged collagen can infiltrate the tissue and cause long-lasting tissue damage (Nathan, 2002; Mahmood et al., 2022). A significant current public health concern is chronic inflammatory diseases (Killeen et al., 2014; Shin et al., 2014). Specific illnesses such as psoriasis, rheumatoid arthritis, periodontal disease, asthma, and atherosclerosis are caused by the dysregulation of inflammatory processes. Additionally, it plays a significant role in the development of several degenerative ailments, including diabetes, Alzheimer’s disease, immunological disorders, and periodontal disease (Libby, 2002; Hansson and Peter, 2006; Sadiq et al., 2018). Additionally, the primary contributor to the harm caused by autoimmune illnesses is the inflammatory response (Jan et al., 2020). As a result, controlling inflammatory processes is a crucial strategy for treating a variety of illnesses. The quest for novel anti-inflammatory substances is still a crucial topic of research, despite the fact that there have been several previous attempts in this direction. This is because conventional medicines employing steroidal or nonsteroidal drugs are frequently associated with ineffectiveness and unfavorable side effects. In the traditional medicine of many cultures, plants have been utilized for generations to treat inflammation-related pain (Alam et al., 2020; Mahnashi et al., 2021). Chronic inflammation causes an overproduction of inflammatory mediators by activating Toll-like receptors found in macrophages. These mediators activate transcription factors that are either directly or indirectly controlled by mitogen-activated protein kinases (MAPKs), including nuclear factor kappa B (NF-kB) and nuclear factor erythroid 2-related factor 2 (Nrf2) (He et al., 2020). Additionally, a number of enzymes are activated, including COX-2 (cyclooxygenase), I kappa B kinase (IKK), inducible nitric oxide synthase (iNOS), and 5-LOX (lipoxygenase) (Ko et al., 2013; Tambuwala, 2016).

An endogenous enzyme called cyclooxygenase (COX) is primarily responsible for turning arachidonic acid into prostaglandins. COX-1 and COX-2 are two prevalent isoforms of this enzyme. COX-2 is an inducible enzyme that is expressed in response to an inflammatory stimulus and promotes inflammation, whereas COX-1 is a constitutive enzyme (Munir et al., 2020; Sadiq et al., 2021). Inflammation can be treated effectively by blocking the enzyme COX-2 (Osama et al., 2024). The untreated inflammation causes an increase in free radicals around the swelling area. Furthermore, the increase in temperature of the inflammatory site and even the whole body is due to the inflammatory response. Therefore, the analgesic and anti-inflammatory drugs work side by side due to their common enzymatic targets (Javed et al., 2022). Due to the uncoupling effects on oxidative phosphorylation, anti-inflammatory, analgesic, and antipyretic effects have been related to the suppression of prostaglandin formation. The pro-inflammatory cytokine tumor necrosis factor (TNF), which is released by a variety of cells and has a wide range of cellular effects, is significant (Montgomery and Bowers, 2012; Zelová and Jan 2013). TNF-α has been linked to a variety of disease states in people, including cancer, psychological disorders, and immunological and inflammatory diseases. IL-1 is a different cytokine that primarily promotes inflammation (Fenton, 1992; Alshehri et al., 2024).

Inflammation has typically been treated with steroids, although the use of these medications has increasingly decreased because of their negative effects (Poetker and Reh, 2010). One of the main classes of medications used to treat inflammation is nonsteroidal NSAIDs, which operate on the afflicted tissues by blocking COX, an enzyme involved in the manufacture of prostaglandins (Javed et al., 2021). Celecoxib (**1**), rofecoxib (**2**), and etoricoxib (**3**) are/were some of the very efficient COX-2 inhibitors, and they represent the COX-2 category of medications (Alqahtani et al., 2022). Rofecoxib and etoricoxib had to be taken off the market due to their damage to the gastrointestinal tract and increased risk of cardiotoxicity and hepatotoxicity (Dvorakova and Landa, 2017). Most of the currently used anti-inflammatory drugs, particularly those with established clinical efficacy, such as aspirin and ibuprofen, have an acidic character. It is, therefore, necessary to develop new anti-inflammatory drugs with great therapeutic efficacy and minimal adverse effects.

**FIGURE 1:**
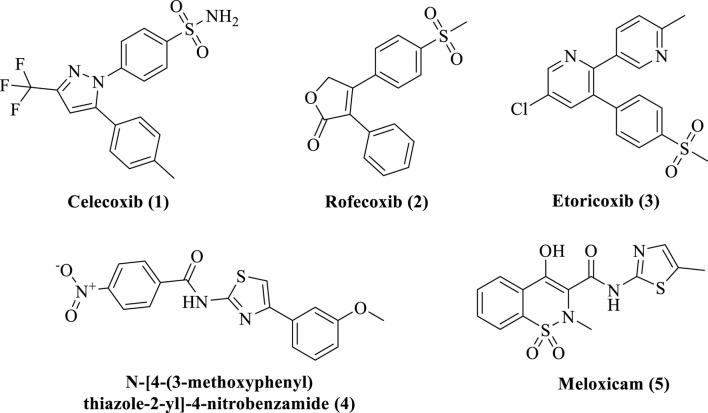
Marketed and reported anti-inflammatory drugs and agents.

The researchers working on organo–medicinal compounds provide effective methods for the development of new drugs, natural products, or building blocks of bioactive compounds (Ahmad et al., 2019; Qayyum et al., 2024). Various synthetic reactions provide baseline structural units for pharmacologically important molecules. Previously, we reported the synthetic approaches for the synthesis of various medicinally important compounds (Bibi et al., 2020; Qayyum et al., 2022). The medicinal compounds, particularly the nitrogenous moieties, have been explored for various medicinal purposes (Nugent et al., 2012; Sadiq et al., 2015; Sadiq and Nugent, 2020; Sadiq et al., 2022). The thiazole nucleus has been regarded as one of the prospective bioactive scaffolds in a variety of biological systems based on the literature that is currently accessible (Shah et al., 2023). Thiazole derivatives have a wide range of medicinal uses, including anti-Alzheimer, anticancer, antibacterial, and anti-diuretic properties. Certain medicines have thiazole rings (Tacconelli et al., 2002; Kovala-Demertzi, 2006). For many years, various natural and synthesized compounds containing the thiazole core moiety have been investigated for their anticancer, antibacterial, anti-HIV, anticoagulant, anti-inflammatory, and antioxidant effects. The bioactivities of thiazole scaffolds are further supported by the literature (Kamat et al., 2020). Anti-inflammatory medications lessen pain by completely or partially halting prostaglandin synthesis (Kawabata, 2011). Well-known anti-inflammatory medications (Figure 1), like meloxicam (**5**), are hybrid substances containing a thiazole core (Kamat et al., 2020). This study has been designed to synthesize new derivatives of thiazole for the treatment of analgesia and inflammation.

## Methodology

### Chemical synthesis

The chemical synthesis of the target compound is a multi-step procedure. The synthesis was initiated by the bromination of 1-(4-chlorothiophen-2-yl)ethan-1-one (**1**, 1.0 equivalent) in the presence of diethyl ether (1.0 M) at room temperature. The brominated product (**2**, 1.0 equivalent) of 1-(4-chlorothiophen-2-yl)ethan-1-one was then treated with thiourea (1.2 equivalent) at a high temperature of 80 C to obtain compound 4-(4-chlorothiophen-2-yl)thiazol-2-amine (**3**). Compound **3**, i.e., 4-(4-chlorothiophen-2-yl)thiazol-2-amine, was then treated with *N*-bromosuccinimide (NBS) (1.0 equivalent) at low temperature (0 C), followed by treatment with primary or secondary amines (1.5 equivalent) to obtain compound **4**. Compound **4** was finally treated with potassium carbonate (0.5 equivalent) in the presence of dimethylformamide (1.0 M) at 70 C to obtain compound **5**.

#### COX-1 activity

The anti-cyclooxygenase-1 activities of the compounds (**5a**–**5g**) were assessed using a modified protocol of the Glassman and White experiment. In brief, the COX-1 enzyme was added to a co-factor solution containing glutathione, hematin, and *N*, *N*, *N*-tetramethyl-p-phenylenediamine dihydrochloride (TMPD) prepared in a Tris-HCl buffer (pH 8.0). Then, test samples were added to the COX-1 enzymes and co-factor solution mixture. After 5 min, arachidonic acid was added and the solution was incubated. After incubation, the absorbance of the final reaction was measured at 570 nm using a UV spectrophotometer. Aspirin was used as a positive control for cyclooxygenase-1 enzyme inhibition (Ahmad et al., 2021).

#### COX-2 activity

The anti-cyclooxygenase-2 activities of the compounds (**5a**–**5g**) were assessed using a slightly modified protocol of the Glassman and White experiment. In short, COX-2 enzymes (300 units per ml) were added to a co-factor solution containing glutathione, hematin, and *N*, *N*, *N*-TMPD prepared in a Tris-HCl buffer (pH 8.0). Then, test samples were added to the COX-2 enzymes and co-factor solution mixture. After 5 min, arachidonic acid was added and incubated. After incubation, the absorbance of the final reaction was measured at 570 nm using a UV spectrophotometer. Celecoxib was used as a positive control for cyclooxygenase-2 enzyme inhibition (Jan et al., 2020). The selective COX-2 inhibitors are normally determined using the selectivity index (SI) of the compounds in both COX-1 and COX-2 assays. A greater SI value shows that the compound is a more selective COX-2 inhibitor. The SI of the compounds can be calculated from the IC_50_ values of their respective COX-1 and COX-2 inhibitors as per the procedure.

#### 5-LOX activity

The 5-lipoxygenase inhibitory activities of the compounds (**5a**–**5g**) were evaluated using different concentrations of the compounds against the standard drug zileuton. In brief, a concentration of 10,000 U per ml of the 5-LOX enzyme was used in the experiment. Moreover, linoleic acid was added as a substrate. Afterward, we dissolved different concentrations of test samples in a phosphate buffer (with pH of 6.3), followed by the addition of the 5-LOX enzyme solution. The mixture was then incubated for 5 min at 25°C. After incubation, we added and mixed the linoleic acid solution with it and kept it for 5 min. Finally, the absorbance was measured at 234 nm using a UV spectrophotometer, and the experiment was repeated three times for optimal results (Jan et al., 2020).

### Experimental animals and ethical approval

The experimental animals used in this study were obtained from the breeding house and were used as per the Animal By-Laws of the University of Malakand. The animals were maintained at a controlled temperature, humidity, and a 12-h dark/light cycle. The animals were provided food and water as per the approval of the ethical committee. The experimental animals used in this study were reviewed and approved by the Research Ethics Committee of the Department of Pharmacy, University of Malakand, Pakistan (Ref No. /055A).

### Carrageenan-induced *in vivo* inflammation

For the *in vivo* anti-inflammatory test, the potential compounds (**5d** and **5e**) were put to the test with carrageenan in albino mice that weighed 25–30 g. In total, there were 5 groups of 40 mice. Each group had eight animals. For group I, the negative control was 10 mL of 1% DMSO per kg of body weight. Group II was the positive control and received 100 mg of aspirin per kg of body weight. Compounds **5d** and **5e** were administered to groups III, IV, and V at amounts of 5, 10, and 20 mg/kg, respectively. Every 30 min, a newly prepared saline solution (0.05 mL) with 1% carrageenan was applied to the sub-planter surface of each mouse. A plethysmometer (LE 7500 Plan Lab SL) was used to measure the paw edema volume every 1–5 h after carrageenan was given. We measured the paw edema of the tested samples and the usual medicine at different times and compared them with the negative control group (Jan et al., 2020). The following method was used to obtain the percentage inhibition of inflammation:

$$\%\text{ inhibition}=EC-\frac{ET}{EC}\times 100,$$

where “EC” is the average edema of the control group and “ET” is the edema of the group that was measured.

### Mechanism of inflammation of the potent compounds

The potent synthesized compounds (**5d** and **5e**) might help reduce inflammation by using paw edema tests that were triggered by prostaglandin E_2_, bradykinin, leukotriene, and histamine. It was done by injecting 10% DMSO, a Bradykinin inhibitor (HOE 140) at a dose of 1 mg/kg, a lipoxygenase inhibitor (montelukast) at a dose of 25 mg/kg, an antihistamine at a dose of 100 mg/kg, the tested compound at a dose of 100 mg/kg, or a cyclooxygenase inhibitor (celecoxib) at a dose of 50 mg/kg. After 1 h, doses of prostaglandin E_2_ (0.01 mg/mL), leukotriene (10 mg/mL), bradykinin (20 mg/mL), or histamine (0.1 mg/mL) under the planter caused paw swelling. The volume of the paw of each mouse was measured immediately before and after irritants (inflammatory agents) were injected under the planter at 1–5 h (Jan et al., 2020).

### Hot plate test

All of the animals were used to being in the laboratory for at least 2 h before the experiments began. An analgesiometer was used to test how well the painkillers worked on a hot plate (Shah et al., 2012). It was kept at 54.0 ± 0.1°C on the hot plates. The animals were given a pre-test, and those with a delay time of less than 30 s were chosen. After the animals were chosen, they were divided into different groups, such as the normal saline group, the standard group, and other groups that would be tried on more powerful samples. Normal water, a standard drug, and different doses of the test sample were all given via abdominal administration. It was possible to determine the duration for which the animals remained on the hot plates at various time intervals.

### Docking study

To investigate the anti-inflammatory inhibition potential of our newly synthesized 4-(4-chlorothiophen-2-yl) thiazole-based compounds, all the compounds were docked deep inside the receptors of the target proteins using Molecular Operating Environment (MOE) software. The crystal structures of the target proteins COX-2 and 5-LOX were retrieved from the Protein Data Bank (PDB) using the four-letter alphanumeric access codes 1CX2 and 6N2W, respectively (Sadiq et al., 2021). All compounds and target proteins were protonated, minimized, and prepared according to the previously reported standard procedure. The docking protocol was verified by the redocking method with RMSD >2.0. After docking calculations, all the orientations were evaluated based on the binding energy of the protein–ligand complex (ΔE = S-score) and the non-covalent interaction deep inside the pocket of target proteins.

## Results and discussion

### Chemistry portion

**SCHEME 1:**
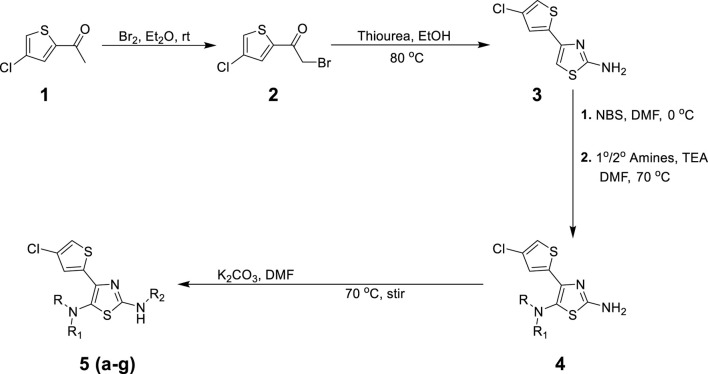
Chemical synthesis of 4-(4-chlorothiophen-2-yl)thiazol-2-amine (**5**).

**FIGURE 2:**
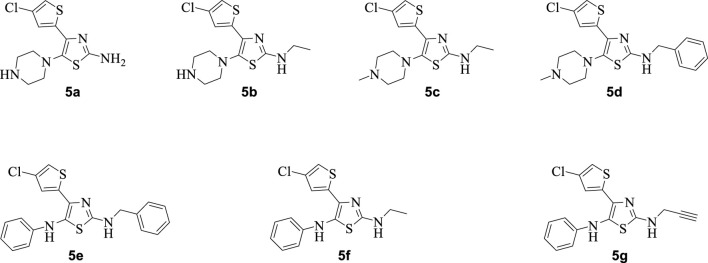
Structures of the synthesized compounds **5a**–**5g**.

The synthetic steps for approaching the target compounds (**5a**–**5g**) are summarized in Scheme 1. Initially, we brominated 1-(4-chlorothiophen-2-yl)ethanone (**1**) by adding bromine (Br_2_) in the presence of ether for 2 h to produce the brominated product 2-bromo-1-(4-chlorothiophen-2-yl)ethan-1-one (**2**). The condensation of 2-bromo-1-(4-chlorothiophen-2-yl)ethan-1-one (**2**) with thiourea produced 4-(4-chlorothiophen-2-yl)thiazol-2-amine (**3**) in 5 h. Compound **3** was then brominated by reacting with NBS, and then, by nucleophilic reaction, the intermediate product was treated with amines to obtain compound **4**. The combined reaction was completed within 24 h. The intermediates produced in the previous step were treated with K_2_CO_3_ to obtain the target products **5a**–**5g** in 24 h, depending on the product. The combined yields for the synthetic steps were in the range of 45%–59%. The structures of the synthesized compounds (**5a**–**5g**) are shown in Figure 2.

### *In vitro* COX/LOX results

The *in vitro* results of cyclooxygenases (COX-1 and COX-2) and lipoxygenase (5-LOX) of the compounds (**5a**–**5g**) are shown in Table 1. Compared to standard zileuton, our compounds (**5a**–**5g**) were less potent as inhibitors of 5-lipoxygenase.

**TABLE 1:** *In vitro* cyclooxygenase inhibition results.

Compound/std.	IC_50_ (μM) ± SEM			Selectivity index (SI)
	5-LOX	COX-1	COX-2	
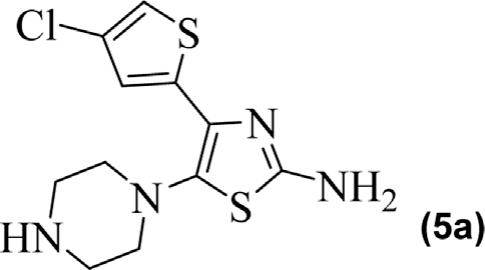	93.15 ± 0.07	52.38 ± 0.04	4.21 ± 0.09	52
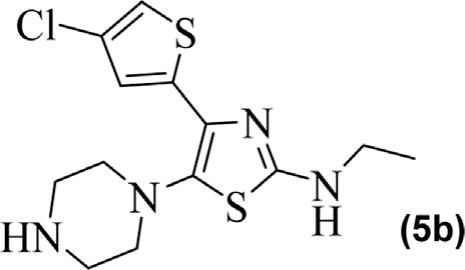	98.26 ± 0.08	39.64 ± 0.10	0.93 ± 0.11	42
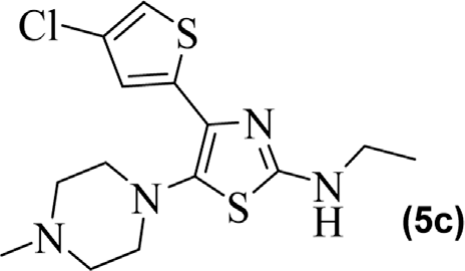	72.32 ± 0.11	66.18 ± 0.18	1.98 ± 0.18	33
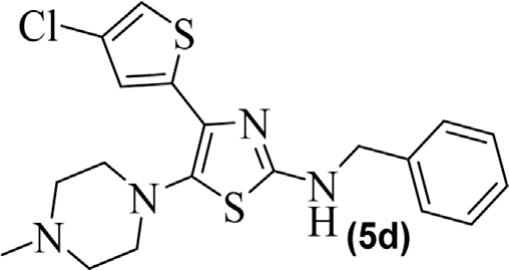	23.08 ± 0.18	93.41 ± 0.06	0.83 ± 0.03	112
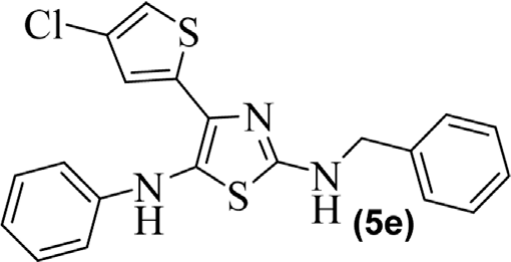	38.45 ± 0.20	94.33 ± 0.14	0.76 ± 0.17	124
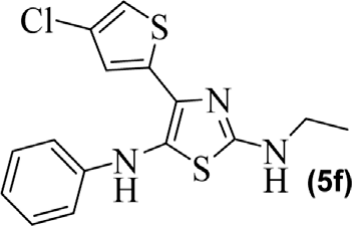	56.21 ± 0.15	34.09 ± 0.13	9.01 ± 0.10	4
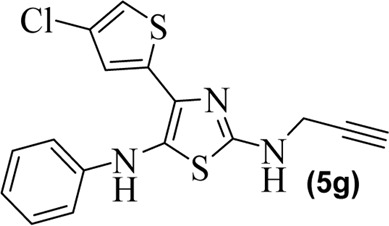	83.11 ± 0.16	25.81 ± 0.12	6.37 ± 0.07	4
Zileuton	11.00 ± 0.12	—	—	—
Aspirin	—	15.32 ± 0.17	—	—
Celecoxib	—	—	0.05 ± 0.01	-

The observed IC_50_ value of zileuton in 5-LOX inhibition was 11.00 μM. Among the compounds, **5d** had the highest activity, showing IC_50_ of 23.08 μM. The 5-LOX inhibition potency of compound **5d** was almost equal to half of that of standard drug zileuton. All the other compounds exhibited IC_50_ values in the range of 38.45–98.26 μM for 5-LOX inhibition. Compared to 5-LOX, our compounds (**5a**–**5g**) were much more potent inhibitors of COX pathways. The leading COX-1 inhibitors were **5b**, **5f**, and **5g** with IC_50_ values of 39.64, 34.09, and 25.81 μM, respectively. All our compounds were very dominant and significant inhibitors of COX-2. The observed range of IC_50_ values was from 0.76 to 9.01 μM. Among the compounds, **5d** and **5e** were the most potent inhibitors of COX-2, with IC_50_ values of 0.83 and 0.76 μM, respectively. Similarly, compound **5b** was also dominant with an IC_50_ value of 0.93 μM. The selectivity index is one of the major parameters for cyclooxygenase inhibitors. A greater value of SI shows that the compound is a more selective inhibitor of COX-2. Among our compounds, **5d** and **5e** had the highest SI values of 112 and 124, respectively. Based on the potencies of these compounds (**5d** and **5e**) and their selectivity indexes, we further used these two compounds for *in vivo* experiments.

### *In vivo* anti-inflammatory activity

#### Carrageenan-induced inflammation results

In carrageenan-induced inflammation, the anti-inflammatory action of the synthesized compounds **5d** and **5e** produced was remarkable. Compound **5d** at the doses of 5, 10, and 20 mg/kg body weight exhibited good activity with percentage inhibition of 45.47 ± 1.11, 49.68 ± 1.44, and 53.44 ± 2.22 at 1 h and was still significant (^***^ *p* < 0.001) up to 5 h with percentage inhibition of 55.34 ± 1.12, 58.56 ± 1.52, and 61.64 ± 1.10, respectively. Likewise, compound **5e** demonstrated the greatest anti-inflammatory activity at doses of 5, 10, and 20 mg/kg. Compound **5e** has outstanding anti-inflammatory activity (55.29% ± 1.04%) at a maximum dose (20 mg/kg) at 1 h, which peaked at 5 h (64.59% ± 1.49%) after carrageenan insertion and remained significant (^***^ *p* < 0.001) throughout investigated drug administration, as shown in Table 2. At a dose of 50 mg/kg for 5 h, aspirin standard medication had a significant effect (59.60 1.52%, ^***^ *p* < 0.001), which was roughly half of the effects caused by compound **5e** at a dose of 20 mg/kg.

**TABLE 2:** Percentage inhibitions of the synthesized compounds in carrageenan-induced paw edema.

Treatment	Dose (mg/kg)	Percent inhibition of paw edema				
		1 h	2 h	3 h	4 h	5 h
Vehicle	--------	7.720 ± 2.70	7.690 ± 1.42	9.40 ± 1.20	10.42 ± 1.52	7.90 ± 2.20
Aspirin	50	48.52 ± 1.12^***^	54.63 ± 1.47^***^	55.22 ± 1.38^***^	58.92 ± 1.95^***^	59.60 ± 1.52^***^
**5d**	5	45.47 ± 1.11^***^	48.10 ± 1.42^***^	51.78 ± 2.10^***^	52.11 ± 2.67^***^	55.34 ± 1.12^***^
	10	49.68 ± 1.44^***^	52.23 ± 1.65^***^	54.45 ± 1.47^***^	55.56 ± 1.52^***^	58.56 ± 1.52^***^
	20	53.44 ± 2.22^***^	55.65 ± 2.21^***^	57.33 ± 1.31^***^	56.89 ± 1.29^***^	61.64 ± 1.10^***^
**5e**	5	47.70 ± 1.68^***^	51.47 ± 1.50^***^	54.56 ± 1.34^***^	53.35 ± 1.47^***^	56.25 ± 1.65^***^
	10	52.36 ± 2.26^***^	54.58 ± 2.58^***^	57.59 ± 1.59^***^	56.78 ± 2.66^***^	62.65 ± 2.79^***^
	20	55.29 ± 1.04^***^	57.39 ± 1.69^***^	60.76 ± 2.68^***^	59.08 ± 1.98^***^	64.59 ± 1.49^***^

The percentage of carrageenan-induced paw edema model in mice was blocked by tested molecules at doses of 5, 10, and 20 mg/kg. The mean (±SEM) for a group of eight mice is shown by each percentage point. Two-way repeated measures ANOVA and Bonferroni’s *post hoc* test were used to analyze the data. Asterisks indicate numbers that are important from the control (car). *p < 0.05, **p < 0.01, and ***p < 0.001; n.s. stands for “not significant.” There are eight mice in each group.

#### Anti-inflammatory mechanism of the potent synthesized compounds 5d and 5e

**FIGURE 3:**
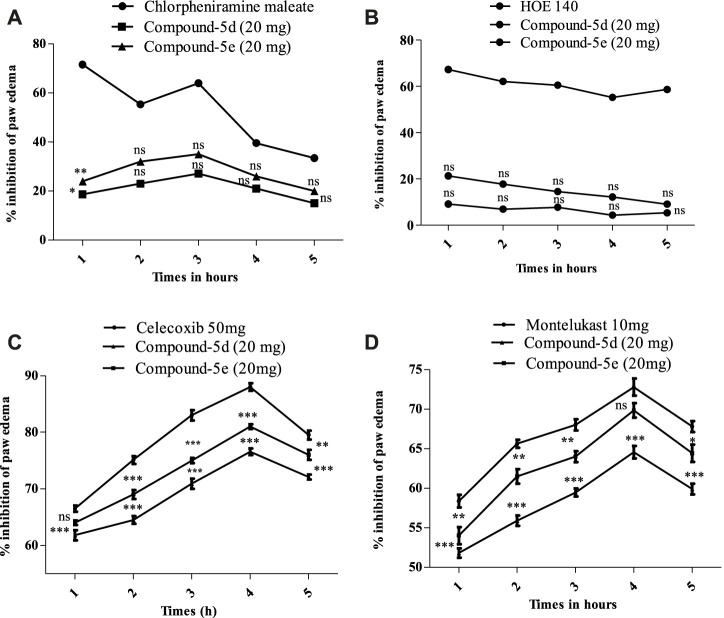
**(A)** Percentage of blockage caused by histamine and compounds **5d** and **5e** (20 mg/kg) in a mouse model of histamine-induced paw edema. **(B)** Amount of blockage caused by bradykinin and compounds **5d** and **5e** (20 mg/kg) in a mouse model of bradykinin-induced paw edema. **(C)** Amount of inhibition that the studied compounds **5d** and **5e** (20 mg/kg) created in a mouse model of prostaglandin E_2_-induced paw edema. **(D)** Amount of reduction that tested compounds **5d** and **5e** (20 mg/kg) caused in a mouse model of leukotriene-induced paw edema. The mean (±SEM) for a group of eight mice is shown by each percentage point. ANOVA and Dunnett’s test were used to analyze the data. *p < 0.05, **p < 0.01, and ***p < 0.001 are all significant, and n.s. means that there was no difference between the experimental and normal drugs.

After observing potential anti-inflammatory effects *in vitro* and *in vivo*, we further investigated the synthesized compounds **5d** and **5e** to understand how they work to fight inflammation. We used different phlogistic agents, such as histamine, bradykinin, prostaglandin, and leukotriene, to test the process. It was found that compound **5d** had a slight antihistaminic effect (27.12%) at the highest dose (20 mg/kg) at 3 h. This may be because it stopped mast cells from releasing mediators. In the same way, compound **5e** showed 35% suppression at 3 h. In addition, the standard drug chlorpheniramine maleate greatly reduced swelling observed from the first to the fifth hour, resulting in a reduction of 71.62%–64.02% caused by histamine (Figure 3A). In the second part, swelling in the mouse paw reached its peak 60 min after the injection of bradykinin. The swelling of the mouse paw was not alleviated by compound **5d**. At the highest doses (20 mg/kg), compound **5d** stopped paw swelling by 9.20% in the first hour. It then decreased but not as much as the normal drug (HOE 140). Similarly, compound **5e** did not yield excellent results either. Its effect peaked at 21.30% in the first hour and then decreased from the second to the fifth hour, as shown in Figure 3B. Figure 3C shows a notable increase in the amount of prostaglandin E_2_ in the paw tissue after the injection of the prostaglandin E_2_ mediator. When mice were given compound **5d** (20 mg/kg) or celecoxib (50 mg/kg), the level of increased PGE_2_ significantly decreased. At the fourth hour, compound **5d** had the strongest effect (76.56 ± 0.58), about the same as a normal drug. It was still significant (***p < 0.001) at the fifth hour. In the same way, compound **5e** was stronger than compound **5d** and showed a suppression of 66.50% ± to 88.0% ± 0.66% from the first to the fourth hour. At the 4th hour after inflammogen, the usual drug celecoxib showed an 88% reduction. Furthermore, leukotriene caused inflammation, and the mouse paws swelled up after it was applied. The swelling reached its highest point 30 min after it was applied. It was found that compound **5d** was very effective at treating mouse paw edema. Compound **5d** showed good results, causing a percentage inhibition of 51.80 ± 0.58, 55.90 ± 0.66, 59.46 ± 0.52, 64.56 ± 0.78, and 59.90 ± 0.68. The compound **5d** result was significant (**p < 0.01) at the first hour and reached its highest level at the fourth hour after the leukotriene mediator was given. It remained significant (***p < 0.001) until the fifth hour. Similarly, compound **5e** showed excellent results, showing 69.84% inhibition at the fourth hour. At the fourth hour (Figure 3D), the usual drug montelukast showed a percentage reduction of 72.79 ± 1.10.

#### Evaluation of anti-nociceptive activity on the hot plate model

The results of the hot plate method of analgesia are presented in Table 3. By the administration of the positive control group after 30 min, the delay time was 17.23, and the painkilling effect was 47.59% ± 0.71% at 100 mg/kg body weight. Synthesized compound **5e** changed the delay time by 14.33 ± 0.44 (*p* < 0.01) and stopped thermal shocks 36.99% ± 0.94% of the time at a dose of 20 mg/kg I/p. Furthermore, compound **5d** greatly reversed the effect, stopping 29.62% ± 0.84% (*p* < 0.05) of the activity at a dose of 20 mg/kg (Table 3). Similarly, after 60 min, the delay time was significantly longer in animals that had been given the usual drug (5 mg/kg tramadol I/P) than in those that had been given the vehicle control group (*p* < 0.001). At a dose of 20 mg/kg I/p, compound **5e** significantly changed the pain-relieving effect, stopping 44.16% ± 0.70% of it (*p* < 0.01). Synthesized compound **5d** that was tested had a delay time of 14 s and blocked the good effects of heat stimuli with a significance level of 35.50% ± 0.50% (*p* < 0.05) at a dose of 20 mg/kg (Table 3). In addition, the latency time was significantly longer in animals that were given the usual drug (5 mg/kg tramadol I/P) than in those given the vehicle control group (latency time 13.27 s; *p* < 0.001). The pain effect was greatly offset by compound **5e**, which had a delay time of 11.25 (*p* < 0.01) and caused percentage analgesic inhibitions of 22.93 ± 0.60 at a dose of 20 mg/kg I/p. Compared to the negative control at the same amount, compound **5d** had a delay time of 8.8 and had a lesser effect than compound **5e**.

**TABLE 3:** Analgesic activity of the synthesized compounds assessed using the hot plate method.

Treatment	Dose (mg/kg)	Latency time in seconds (mean ± SEM)		
		After 30 min	After 60 min	After 90 min
Normal saline	10 mL/kg	8.01 ± 0.22	9.03 ± 0.28	7.55 ± 0.59
Standard	5 mg/kg	16.31 ± 0.47^***^	18.50 ± 0.58^***^	12.25 ± 0.31^***^
**5d**	20 mg/kg	12.83 ± 0.52^*^	14.00 ± 0.10^***^	8.83 ± 0.80^*^
**5e**	20 mg/kg	14.33 ± 0.44^**^	16.17 ± 0.42^**^	11.25 ± 0.52^**^

The information shows pain-relieving effects using a hot plate test on mice. The results are shown as the mean ± SEM (*n* = 6) and were analyzed using a two-way ANOVA, followed by Bonferroni’s *post hoc* test. ∗M < 0.05; ∗∗*p* < 0.01, and ∗∗∗*p* < 0.001; ns; not important.

### Docking studies

#### Docking studies on COX-2

The structure of COX-2 was retrieved from the protein database with PDB ID 1CX2. It contains the COX-2 enzyme complexed with the native ligand (4-[3-(trifluoromethyl)-5-(4-bromophenyl)-1H-pyrazol-1-yl]benzenesulfonamide). The native ligand displayed important interactions with amino acid residues, including His90, Leu352, Ser353, Arg513, Tyr355, Arg120, Val349, Val523, Tyr385, Trp387, Phe381, Leu384, and Ala529.

Compound **5d** displayed important binding affinities similar to those of native ligands, including vital hydrophilic hydrogen bond interactions with His90 and Ser353 through the hydrogen of the NH group. It also displayed pi–sulfur interaction (hydrophobic) with Tyr355 and Phe518 by the sulfur of the thiazole and thiophene regions. Other hydrophobic interactions include the π–π stacked interaction with Asp515 and the π–alkyl interaction with Val523.

**FIGURE 4:**
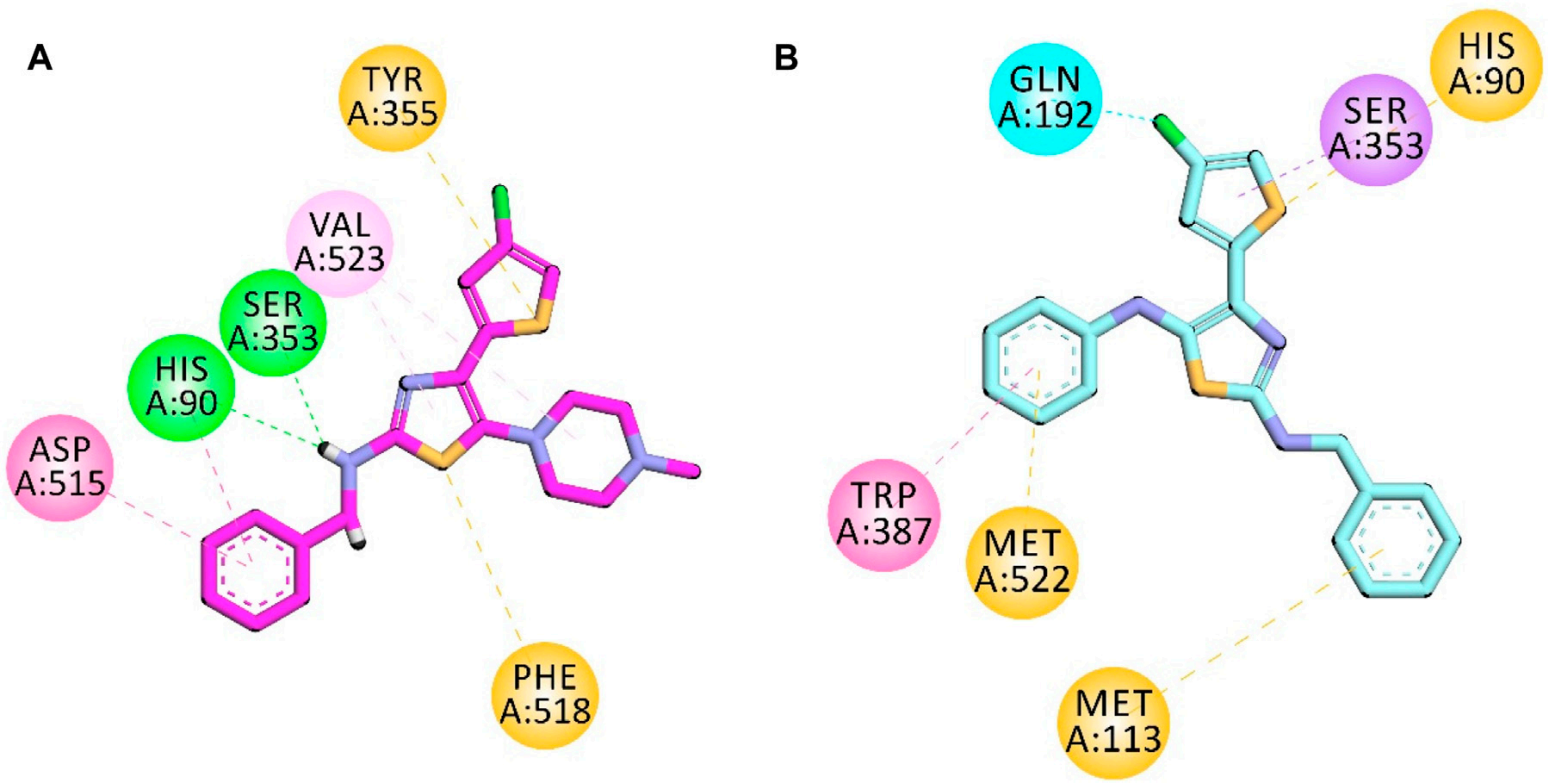
2D interaction diagram of synthesized compounds **(A) 5d** and **(B) 5e** in the binding site of COX-2 (PDB ID = COX2).

Compound **5e** also displayed binding affinities with important residues similar to the native ligand. It includes pi–sulfur hydrophobic interaction with His90 through the sulfur of the thiophene region. The aromatic region in the benzyl region showed pi–sulfur interaction with Met522 and Met113. Pi–sigma interaction was also displayed between the Ser353 amino acid residue and the aromatic region of thiophene. The halogen attached to the thiophene group also displayed interaction with Gln192. The aromatic region of one terminal side of the molecule displayed binding affinity (hydrophobic interaction) with Trp387 (Figure 4).

#### Docking studies on 5-LOX

The co-crystalized structure of enzyme 5-LOX having native ligand nordihydroguaiaretic acid (NDGA) was obtained from the PDB having a code 6N2W. The native ligand displayed binding affinities with the following amino acid residues: His600, Leu607, Arg596, Asn407, Phe359, Trp599, His372, His367, and Ala410.

Compound **5d** displayed binding affinities with amino acid residues similar to those of native ligands, including hydrophobic interactions with His367, Trp599, and Phe359 (pi–pi T-shaped) through the thiophene aromatic region and aromatic region present at the one terminal end. The halogen group attached to the thiophene ring displayed halogen interactions. Pi–sulfur hydrophobic binding affinity was displayed with the His372 residue through the sulfur of the thiophene group. Other hydrophobic binding affinities include Leu607 and Ala410.

**FIGURE 5:**
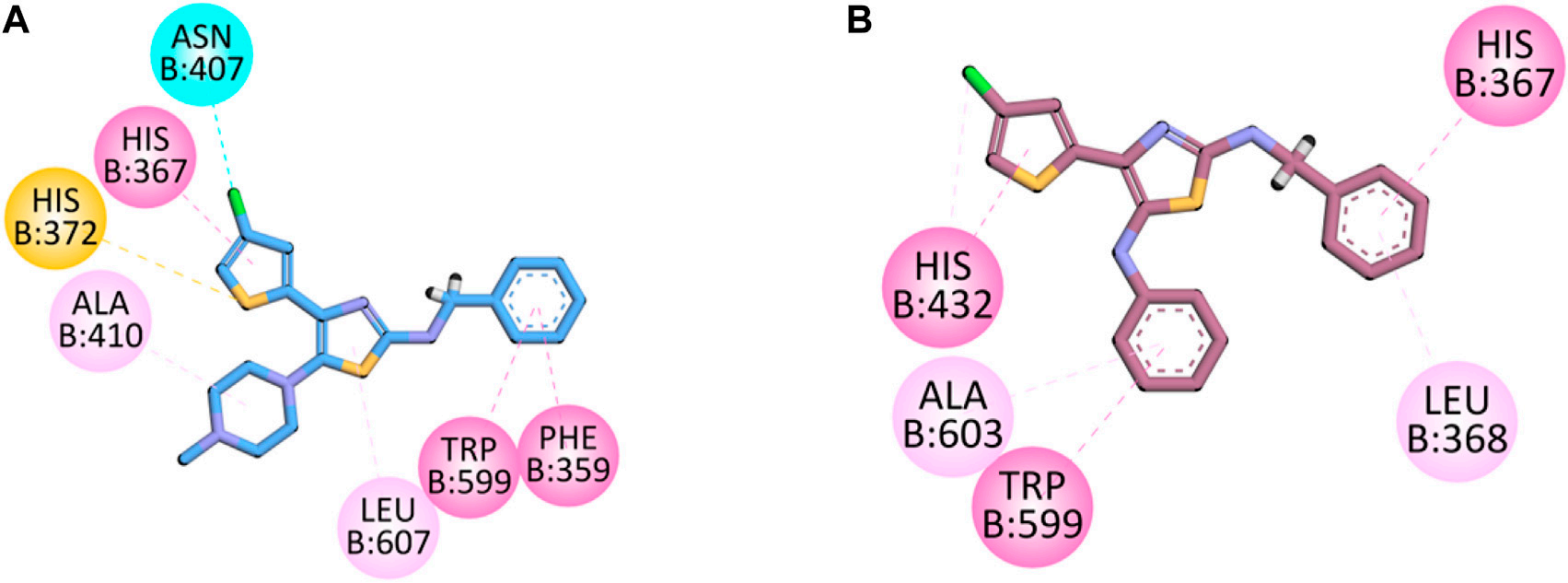
2D interaction diagram of synthesized compounds **(A) 5d** and **(B) 5e** in the binding pocket of 5-LOX (PDB ID: 6N2W).

Compound **5e** also displayed binding affinities similar to those of native ligands, including hydrophobic interactions with His367 (pi–pi T-shaped), His 432, and Trp599 (pi–pi stacked) with an aromatic region present on both terminals of the synthesized molecule and thiophene aromatic region. It also displayed pi–alkyl hydrophobic interactions with Leu268 and Ala603 (Figure 5).

Analgesia and its major cause, inflammation, are medical complications that go side by side in many ailments (Zhang and An, 2007; Mahnashi et al., 2021). Both of these ailments are protective responses to injuries and external stimuli (Antonelli and Kushner, 2017). Hotness, or analgesia, is among the major symptoms of inflammation. The current marketed analgesic and anti-inflammatory drugs are associated with severe unwanted effects (Carter et al., 2014). To minimize toxicity and improve the activity profile, medicinal chemists synthesize various potent molecules for the treatment of analgesia and inflammation (Zayed, 2022). Previously, we synthesized simple pyrrolidinedione and other Michael products as potential analgesic and anti-inflammatory agents (Ahmad et al., 2021; Sadiq et al., 2021). In this research, we found that bis-succinimide efficiently inhibits the COX/LOX pathway for the management of analgesia and inflammation.

## Conclusion

In this study, we synthesized 7 new 4-(4-chlorothiophen-2-yl)thiazol-2-amine derivatives (**5a**–**5g**) as potential anti-inflammatory and analgesic agents. The compounds were synthesized efficiently through multistep chemical reactions. The pharmacologically important building blocks of thiazoles, specifically, compounds **5d** and **5e**, have been proven to be potential inhibitors of COX/LOX pathways. Compared to the respective standard drugs (zileuton for 5-LOX, aspirin for COX-1, and celecoxib for COX-2), compounds **5d** and **5e** were potent anti-COX/LOX agents. Moreover, our compounds also proved to be safe in experimental animals. The compounds proved to be very active analgesic and anti-inflammatory agents in *in vivo* models. The molecular docking of the selected compounds in the enzymes COX-1, COX-2, and 5-LOX further supported our results and confirmed that our synthesized compounds effectively bind to the amino acid residues of the target site. In this study, we explored the thiazole scaffold as a potential analgesic and anti-inflammatory building block.

## Data Availability

The original contributions presented in the study are included in the article/Supplementary materials, further inquiries can be directed to the corresponding authors.
